# Inadequate labeling of pork sausages prepared in Corsica causing a trichinellosis outbreak in France

**DOI:** 10.1051/parasite/2016027

**Published:** 2016-06-17

**Authors:** Caroline Ruetsch, Pascal Delaunay, Alexis Armengaud, Françoise Peloux-Petiot, Jean Dupouy-Camet, Isabelle Vallée, Bruno Polack, Pascal Boireau, Pierre Marty

**Affiliations:** 1 Parasitologie-Mycologie, Hôpital de l’Archet, Centre Hospitalier Universitaire de Nice 06200 Nice France; 2 Inserm U1065, Centre Méditerranéen de Médecine Moléculaire, Université Nice-Sophia Antipolis 06000 Nice France; 3 Cellule de l’Institut de Veille Sanitaire en régions Provence-Alpes-Côte d’Azur et Corse 13331 Marseille France; 4 Agence Régionale de Santé de Provence-Alpes-Côte d’Azur 06202 Nice France; 5 Parasitologie-Mycologie, Hôpital Cochin, Assistance Publique Hôpitaux de Paris 75014 Paris France; 6 ANSES, ENVA, INRA, Université Paris-Est, Laboratoire de Santé Animale ANSES, UMR BIPAR 94700 Maisons-Alfort France

**Keywords:** Trichinellosis, *Trichinella*, Sausage, *Figatelli*, Pork, Corsica, France

## Abstract

Three cases of human trichinellosis due to *Trichinella britovi* were reported in 2015 in the Southeast of France resulting from consumption of raw pork sausages (*figatelli*) prepared in Corsica. Fourteen other people ate *figatelli* from the same batch but were not infected due to the *figatelli* being well cooked. This is the first reported human trichinellosis outbreak due to consumption of Corsican sausages prepared from uncontrolled pork. Consumption of raw *figatelli* is a common tradition in Corsica. As a result, the health recommendation to cook the product well is not always applied. In the present case, the *figatelli* product label was not sufficiently visible to advise consumers of the risks associated with uncooked pork.

## Abstract (fr)

Trois cas de trichinellose humaine due à *Trichinella britovi* ont été rapportés en 2015 dans le Sud-Est de la France après consommation de saucisses de porc crues (*figatelli*) préparées en Corse. Quatorze autres personnes ont mangé des *figatelli* de ce même lot, mais aucune n’a été infectée car la cuisson des *figatelli* était à cœur. Nous rapportons la première épidémie de trichinellose humaine due à la consommation de saucisses corses préparées à partir de porc non contrôlé. La consommation de *figatelli* crue est culturellement ancrée en Corse, et la recommandation d’une cuisson suffisante n’est pas toujours suivie. En effet, la taille de l’étiquette des *figatelli* était insuffisante pour prévenir les consommateurs des risques associés au porc insuffisamment cuit.

## Introduction

Trichinellosis is a worldwide zoonosis caused by parasitic nematodes of the genus *Trichinella*, a parasitic worm widespread globally in wildlife and frequent regionally in domestic pigs [7]. Human infections are most common in cultures where dietary habits include consumption of raw or undercooked meat. Trichinellosis can be a serious human disease, particularly in elderly individuals, in whom severe complications such as myocarditis or encephalitis can lead to death [3]. The diagnosis of human trichinellosis is based on a set of clinical and biological symptoms associated with epidemiological features. It should be suspected in the presence of a clinical triad including fever, myalgia, and facial edema, associated with high blood eosinophil counts and elevated serum levels of muscle enzymes [3]. The consumption of raw or undercooked meat from game in the previous weeks should be investigated. The diagnosis is confirmed by a positive result for specific serological tests detecting *Trichinella* antibodies (ELISA, western blot). The identification of larvae in a muscle biopsy from the patient is unnecessarily traumatic and not always conclusive, and the parasite can sometimes be detected in remaining contaminated meat. Wild animals act as a reservoir for three species in France: *Trichinella spiralis*, *T. britovi* (found more commonly in mountain regions) and more rarely, *T. pseudospiralis* [10]. In France, 36 outbreaks of trichinellosis occurred between 1975 and 2011 causing 2497 human infections (National Reference Centre for *Trichinella*). Most of the cases were associated with eight outbreaks related to horsemeat consumption, occurring between 1979 and 1998. Wild boar meat consumption was the source of trichinellosis in 146 cases reported in 24 outbreaks [2, 3], whereas the domestic pig was responsible for only one human infection [4]. This occurred in the South East of France and involved 21 patients who consumed pork from a backyard pig fed on fox carcasses.

Nowadays in France, autochthonous trichinellosis is a well-controlled parasitosis, as only two cases were identified between 2011 and 2014 (Department of Parasitology, Cochin Hospital, in charge of surveillance of human cases in France since 2012). We report an outbreak that occurred in March 2015 in patients living in the South of France (region of Nice) after they consumed raw *figatelli*, a Corsican sausage bought in Corsica and delivered by post to mainland France.

## Case report

On 5 February 2015, a 59-year-old woman living near Nice (France) received 10 *figatelli* ordered a few days before from a delicatessen based in Aullène, a village in Southern Corsica. *Figatelli* sausages are a Corsican specialty prepared from pork products: mainly raw liver, but also raw muscles, heart, and spleen. The woman gave *figatelli* to 16 friends and ate raw *figatelli* herself between 13 and 20 February. On 5 March, she went to the hospital due to high fever (40 °C), severe myalgia, facial edema, and conjunctivitis, and was found to have an eosinophil count of 9000/mm^3^. In the following days, two friends had similar symptoms: a 50-year-old woman who had eaten raw *figatelli* between 23 and 28 February and who was hospitalized on 12 March, and a third person who ate *figatelli* only once (on 27 February) and who had less severe symptoms. Trichinellosis was not originally diagnosed and it was only at the end of March that *Trichinella* serology was ordered. On 3 April, three of the patients had specific anti-*Trichinella* antibodies, as detected by ELISA (Immuno-Biological Laboratories, USA) and immunoblotting (LDBio Diagnostics, France). The three patients were treated with albendazole at a dose of 15 mg/kg for 15 days. One displayed allergic manifestations and required corticosteroids. The outcome was favorable, though visual signs (binocular diplopia, visual discomfort) and residual asthenia lasted for 3 months in two patients. The laboratory signs (abnormal blood eosinophilia and muscle enzymes) rapidly returned to normal after treatment.

**Figure 1.**
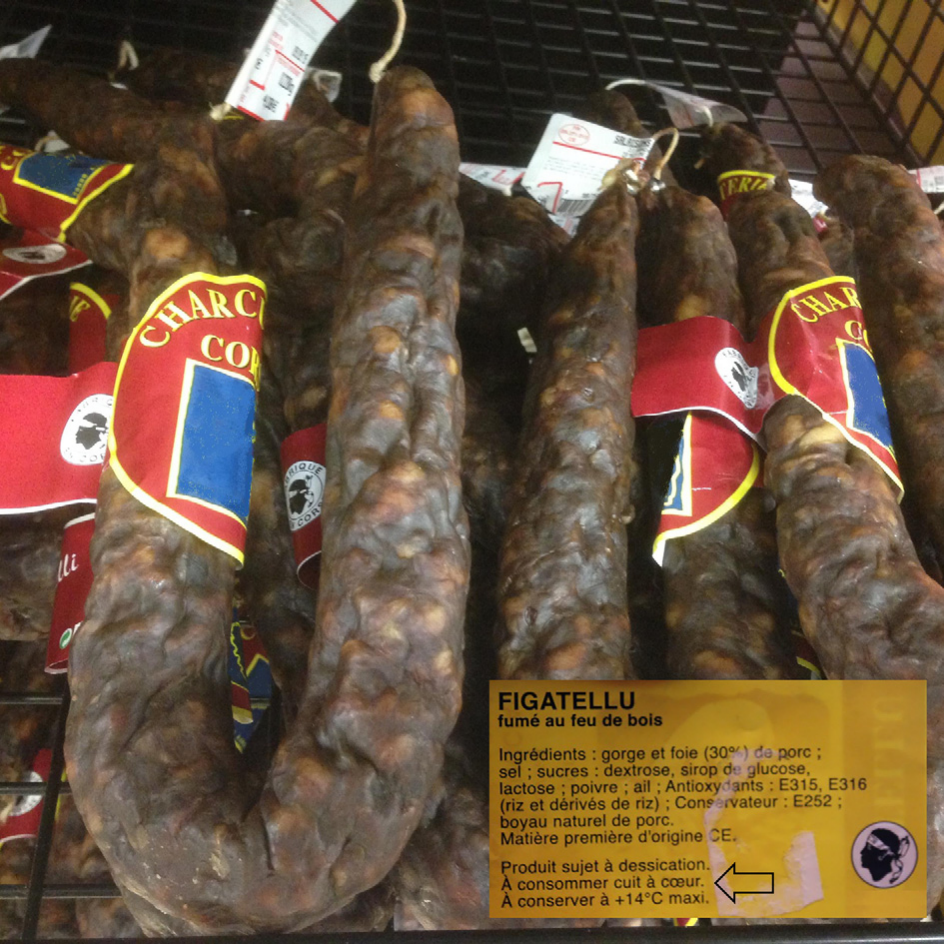
The font size on the *figatelli* label recommending sufficient cooking of the product (“À consommer cuit à cœur” meaning “cook well before eating”) is too small for the warning to be easily read by consumers.

Investigation of the outbreak was carried out by the Regional Health Agency (ARS), which was informed of the foodborne outbreak on 4 April 2015. The patient identified as the index case gave *figatelli* to colleagues, neighbors, and friends without any recommendation on the need for sufficient cooking, though this was written on the label (Fig. 1). In addition to the three affected patients, five different families, comprising 14 people, consumed *figatelli* from the same batch. All exposed people were asymptomatic at the time of examination, did not mention clinical signs or symptoms suggestive of trichinellosis, and all the laboratory tests performed were normal (blood cell counts, muscle enzymes, *Trichinella* antibody testing). All *figatelli* consumed by the 14 exposed people had been well cooked. The Regional Health Agency office in Corsica provided targeted information to consumers and health professionals to raise awareness of the diagnosis of trichinellosis with the suggestive clinical signs and abnormal blood eosinophil counts and emphasized the usual preventive measures.

Veterinary investigation was coordinated by the Laboratory for Animal Health (ANSES) at Maisons-Alfort, France. Three of the ten incriminated *figatelli* sausages had been kept by the index patient and were analyzed by chlorhydropeptic digestion. A *Trichinella* burden of 4 larvae per gram was found in the three sausages. The larvae were typed as *T. britovi* by multiplex PCR performed at ANSES. The Official Veterinary Services of Corsica seized all remaining delicatessen products that had been stored and some were found to be parasitized by *T. britovi* larvae. This particular producer breeds his own pigs and usually sends them to a slaughterhouse in Cozzano (a small village of the Taravo valley, 30 km north of Aullène), where official *Trichinella* control is performed. In this case, the incriminated products were prepared from pigs that had been illegally slaughtered and not controlled for possible *Trichinella* infection.

## Discussion

This small outbreak due to pork consumption is the third reported in France and the first outbreak ever reported after consumption of pork from Corsica. Only two outbreaks related to pork had previously been described in France: the 1878 outbreak in Crépy-en-Valois and the 1983 outbreak in South East France [4, 5]. The emergence of human cases in Corsica was not unexpected, as a focus of swine trichinellosis due to *T. britovi* had been identified in Cozzano as early as 2004, when ten infected pigs were identified at the local slaughterhouse [11]. Epidemiological studies conducted on foxes from this region enabled detection of one positive animal out of 74. Interestingly, between 2006 and 2008, no wild boar was found to be positive among 1881 animals tested by muscle digestion [11]. Since then, an accredited local routine veterinary laboratory controls all pigs slaughtered locally. This control led to the detection of positive outdoor pigs: three in 2010, four in 2011, six in 2012, and two in 2013 [12]. Similarly, human cases have been reported in the neighboring island of Sardinia, Italy [8]. In April 2005, an outbreak of 11 cases due to the consumption of infected pork was reported to have occurred in the villages of Orgosolo and Lanusei (Nuoro province). Subsequent investigations showed that a second outbreak occurred in December 2005 and artificial digestion of muscle samples from 681 free-range and backyard pigs revealed *T. britovi* larvae in four sows (0.58%) [9]. For 60 years, the islands of the Mediterranean Basin were considered to be *Trichinella*-free, but the emergence of *Trichinella* infection in Sardinia and Corsica invalidates this concept even though the origin of the infected animals remains to be determined. Recent preliminary results of microsatellite analysis of *T. britovi* isolates from Sardinia, showing genetic differences with both continental and Corsican isolates, suggest different geographic origins and historical introduction of *T. britovi* to Sardinia. On the contrary, the detection in Corsican isolates of alleles circulating in continental Europe could suggest recent introduction of *T. britovi* to this island [6]. In France, human trichinellosis is an endemic but rare foodborne parasitosis. Consequently, consumers and the medical community have limited knowledge of the disease, sometimes leading to delayed diagnosis, as described here. Delayed diagnosis of the present outbreak may explain why in two patients visual signs and residual asthenia lasted for 3 months. Consumption of raw *figatelli* is a cultural tradition in Corsica and the recommendation for sufficient cooking is not always applied. The font size on the *figatelli* label was too small and inadequate to effectively warn consumers (Fig. 1). The recommendation “to cook well before eating” (*À consommer cuit à cœur*) should be more visible. Given that there is also a risk of hepatitis E associated with the consumption of raw figatelli [1], we strongly recommend a change to the product labeling to better inform and thus, protect consumers. Finally, as pigs are extensively bred outdoors in Corsica, pork must imperatively be tested at routine accredited veterinary laboratories by specific methods for detection of *Trichinella* larvae according to EU regulations (Regulation (EC) No. 2075/2005 implemented by Regulation (EU) 2015/1375). In the present case, a temporary lack of official control associated with poor information for non-local consumers caused a failure in parasite control, which resulted in trichinellosis in three patients.

## Conflict of interest

The authors have no potential conflicts of interest to disclose.
